# Activity of the Lateral Hypothalamus during Genetically Determined Absence Seizures

**DOI:** 10.3390/ijms22179466

**Published:** 2021-08-31

**Authors:** Péter Sere, Nikolett Zsigri, Timea Raffai, Szabina Furdan, Fanni Győri, Vincenzo Crunelli, Magor L. Lőrincz

**Affiliations:** 1 Department of Physiology, Anatomy and Neuroscience, Faculty of Sciences University of Szeged, 6726 Szeged, Hungary; peter.sere86@googlemail.com (P.S.); zsigrinikol@gmail.com (N.Z.); timea.raffai8@gmail.com (T.R.); nadruf77@gmail.com (S.F.); fannigyori93@gmail.com (F.G.); 2 Department of Physiology, Faculty of Medicine, University of Szeged, 6720 Szeged, Hungary; 3 Neuroscience Division, Cardiff University, Museum Avenue, Cardiff CF10 3AX, UK; crunelli@cardiff.ac.uk

**Keywords:** epilepsy, spike-wave discharge, serotonin, hypothalamus

## Abstract

(1) Background: Absence seizures (ASs) are sudden, transient lapses of consciousness associated with lack of voluntary movements and generalized 2.5–4 Hz spike-wave discharges (SWDs) in the EEG. In addition to the thalamocortical system, where these pathological oscillations are generated, multiple neuronal circuits have been involved in their modulation and associated comorbidities including the serotonergic system. Neuronal activity in one of the major synaptic input structures to the brainstem dorsal raphé nucleus (DRN), the lateral hypothalamus (LH), has not been characterized. (2) Methods: We used viral tract tracing and optogenetics combined with in vitro and in vivo electrophysiology to assess the involvement of the LH in absence epilepsy in a genetic rodent model. (3) Results: We found that a substantial fraction of LH neurons project to the DRN of which a minority is GABAergic. The LH to DRN projection can lead to monosynaptic iGluR mediated excitation in DRN 5-HT neurons. Neuronal activity in the LH is coupled to SWDs. (4) Conclusions: Our results indicate that a brain area involved in the regulation of autonomic functions and heavily innervating the RN is involved in ASs. The decreased activity of LH neurons during SWDs could lead to both a decreased excitation and disinhibition in the DRN. These results support a long-range subcortical regulation of serotonergic neuromodulation during ASs and further our understanding of the state-dependence of these seizures and some of their associated comorbidities.

## 1. Introduction

Absence seizures (ASs) are sudden, transient lapses of consciousness associated with lack of voluntary movements and generalized 2.5–4 Hz spike-wave discharges (SWDs) in the electroencephalogram (EEG) [1]. ASs in children and teenagers are generally considered benign, but 30% of children with ASs are pharmaco-resistant [2,3] and 60% suffer from various psychiatric comorbidities [4]. SWDs are generated in the thalamocortical system^1^, but neuromodulatory circuits including the serotonergic [5,6,7], dopaminergic [8], cholinergic [9] and noradrenergic [10,11] systems have been suggested to play a role in these non-convulsive seizures.

Serotonin (5-HT) is a central neuromodulator implicated in the regulation of many (patho)physiological functions and one of the most important targets for psychoactive drugs [12,13,14]. Selective serotonin reuptake inhibitors (SSRIs) show an antiepileptic effect^5^ and the activity of neurons in the main serotonergic nuclei, the brainstem raphe nuclei is correlated with seizures. Specifically, 5-HT neurons decrease firing during and after convulsive seizures [15], but show increased activity during SWDs in WAG/Rij rats [16]. Selective activation of 5-HT2aRs or 5-HT2cRs decreases SWDs whereas blocking 5-HT2aRs increases SWDs [17], highlighting the importance of the 5-HT system in controlling ASs.

One of the main inputs to the dorsal raphe nucleus (DRN) is the lateral hypothalamus (LH) [18,19], a brain area consisting of neurochemically heterogeneous neuronal populations implicated in the modulation of arousal [20] and control of energy intake [21]. GABAergic neurons in the LH selectively inhibit thalamic reticular nucleus neurons [22] and DRN GABAergic neurons [23] promoting wakefulness. The LH receives prominent cortical projections from the medial prefrontal and orbitofrontal cortices [24,25,26]. SWDs are present in the local field potential (LFP) recorded in the medial prefrontal cortex of WAG/Rij [16] and GAERS [27] rats and small amplitude SWDs have been recorded in the lateral hypothalamus of Wistar rats [28], but the activity of LH neurons during SWDs has not investigated to date. Using LFP and single unit recordings from the LH during spontaneous SWDs in awake behaving GAERS rats we show for the first time that: (i) SWDs can spread to the LH; (ii) the activity of LH neurons is correlated with the SWDs on various timescales; and (iii) LH neurons directly contact DRN 5-HT neurons. These results have important implications in the pathophysiology of ASs and associated comorbidities.

## 2. Results

### 2.1. Functial Connectivity between LH and DRN

**Figure 1:**
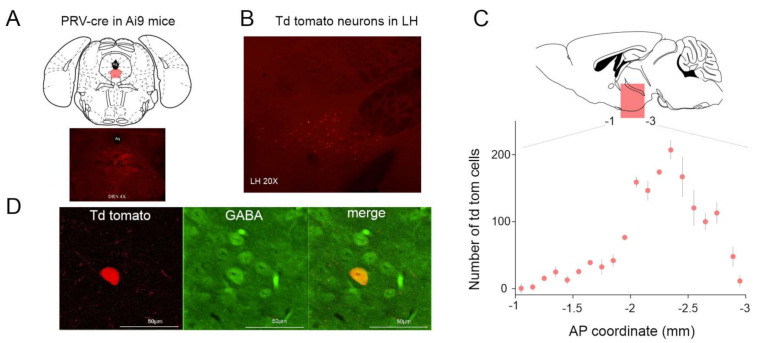
Projections from LH (lateral hypothalamus) to DRN (dorsal raphe nucleus). (**A**) Schematics of the experimental design for retrograde labeling of LH neurons projecting to the DRN. (**B**) Coronal section of a mouse brain at the level of the LH shows tdTomato expression following PRV-cre injection in the DRN. (**C**) Distribution of the tdTomato expressing somata in the LH. AP: anteroposterior. (**D**) Immunohistochemical labeling of GABAergic neurons.

Injection of AAV-syn-ChR2-Venus [29] into the LH resulted in somatic ChR2-Venus expression in the LH region and dense axonal expression in the brainstem area including the DRN [23]. To confirm the results of the anterograde tracing we injected the retrograde viral vector PRV-cre [30] in the DRN area (Figure 1A) of tdTomato reporter mice (Ai9, RCL-tdTomato, Jackson Laboratory, Bar Harbor, ME, USA). Sixty days following the injection tdTomato expressing neurons could be visualized in the LH area (Figure 1B,C). Notably, out of 318 retrogradely labeled neurons tested, 26 were immunopositive for GABA (8.17%, Figure 1D).

**Figure 2:**
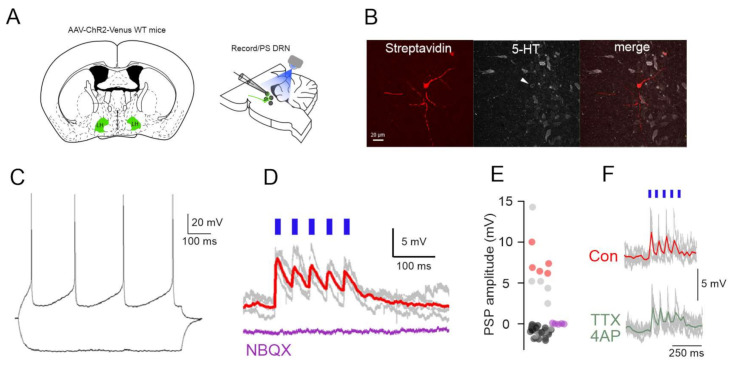
Functional excitatory projections from LH to DRN. (**A**) Schematics of the experimental design for the in vitro circuit mapping experiments. (**B**) Fluorescent images (Streptavidin, 5-HT immunostaining, merged) of a DRN 5-HT neuron responding to LH axonal photostimulation. (**C**) Membrane potential responses of the neuron in (**B**) to hyperpolarizing and depolarizing current steps. (**D**) The photostimulation of LH axons leads to EPSPs in the neuron shown in (**B**,**C**) (gray traces: individual voltage responses, red trace: average) that are blocked by the iGluR antagonist (NBQX, purple trace). (**E**) Distribution of PSP amplitudes in all the recorded neurons (n = 51) (red circles indicate identified DR-5-HT neurons, gray circles unidentified neurons, purple circles EPSP amplitudes following NBQX application (n = 5). (**F**) Persistence of the LH axonal photostimulation induced EPSPs in TTX/4AP in a DRN neuron.

We next used optogenetics to functionally characterize the LH to DRN circuit (Figure 2A). We found that photostimulation of AAV-syn-ChR2-Venus-expressing LH terminals in the DRN evoked excitatory postsynaptic potentials (EPSPs) of 7.58 ± 0.9 mV peak amplitude with a 11.07 ± 2.46 ms decay time in 10 out of 51 neurons tested (20%) DRN cells (Figure 2D,E) and inhibitory postsynaptic potentials (IPSPs) in seven neurons (14%) [23]. Of the 10 neurons showing EPSPs five were confirmed to be DRN serotonergic neurons using post-hoc immunohistochemistry (Figure 2B,E). No detectable PSPs could be recorded in the remaining population (n = 34, 66%, Figure 2E). The EPSPs were completely blocked by the AMPA/KA receptor blocker 50 μM NBQX (n = 5 out of 5 cells, Figure 2D,E). The EPSPs persisted in the presence of TTX and 4-AP (mean EPSP amplitude: control: 4.7 ± 0.9 mV, TTX/4AP: 3.15 ± 0.8 mV, *p* < 0.05, Wilcoxon signed-rank test, n = 5 neurons, Figure 2F) arguing for the monosynaptic nature of the recorded postsynaptic potentials. These results show that the LH to DRN projections are functional and DRN 5-HT neurons receive monosynaptic iGluR mediated EPSPs from LH axons.

### 2.2. Local SWDs in the LH

**Figure 3:**
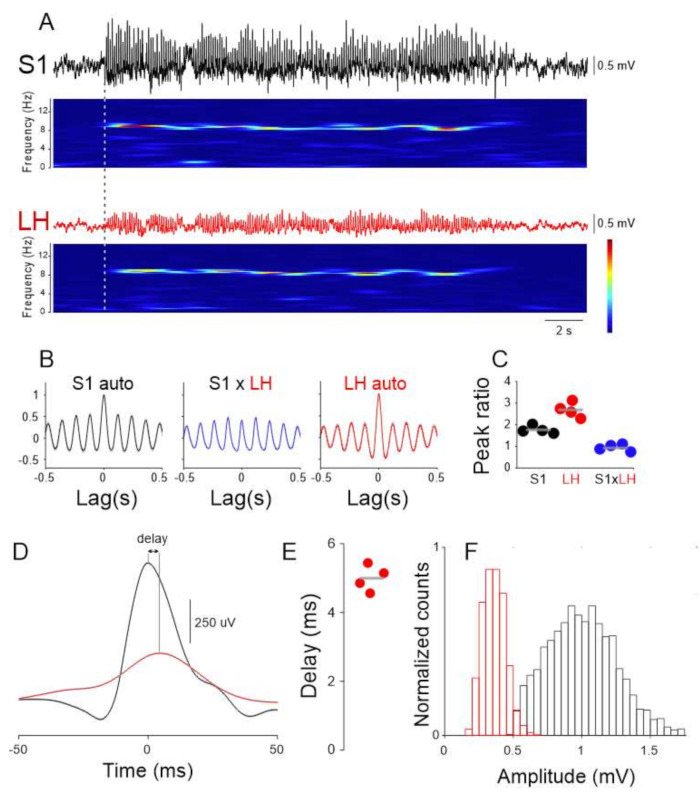
Ictal LFP (local field potential) oscillations in the LH. (**A**) Simultaneous recording of LFPs in the S1 (black trace) and LH area (red trace) and corresponding spectrograms show clear activity at ~9 Hz. (**B**) S1 autocorrelation (left), S1 vs. LH cross-correlation (middle, blue trace) and LH autocorrelation (right, red trace) taken from 20 consecutive ictal episodes. Solid lines represent the mean, gray shaded area: s.e.m. (**C**) Quantification of the main/side peaks for the three correlations from four animals (Color codes as in (**A**,**B**), gray lines indicate the mean). (**D**) SWD peak triggered average of S1 (black) and LH (red) LFP. Solid lines represent the mean, gray shaded area: s.e.m. (**E**) Quantification of the delay between S1 and LH peaks of SWD cycles illustrated in (**D**) in four animals. (**F**) Distribution of the SWD amplitudes in S1 (black) and LH (red).

Given the involvement of the 5-HT system in absence epilepsy and the reciprocal connections between LH and DRN, we investigated whether and how LH neuronal activity is influenced by the generalized SWDs that are the stereotypical signature of ictal periods in a validated rodent model of absence epilepsy, the Genetic Absence Epilepsy Rats from Strasbourg (GAERS) [31]. We simultaneously recorded LFPs from the cortical initiation network (CIN) [32], i.e., the perioral region of the primary somatosensory cortex (S_1_) and the LH in freely moving GAERS rats. SWDs in the LH were of smaller amplitude (S1: 1016.0 ± 6.38 μV, LH: 361.0 ± 2.0 μV, Figure 3A,E), but similar mean frequency than those recorded in S1 (S1: 8.5 ± 0.04 Hz, LH: 7.96 ± 0.4 Hz, *p* > 0.05, Wilcoxon rank-sum test, Figure 3B,C,F). The onset of SWDs in LH always occurred after the S_1_ (mean onset delay: 380.5 ± 28.07 ms, Figure 3A). Quantification of the temporal relationship between individual cycles of the SWDs recorded in S1 and LH showed that those in the latter area were delayed compared to the former area (mean delay: 4.73 ± 0.27 ms, Figure 3D,E). These results suggest that LH neuronal circuits are entrained to the ongoing SWDs.

### 2.3. LH Neuronal Activity Correlates with SWDs

**Figure 4:**
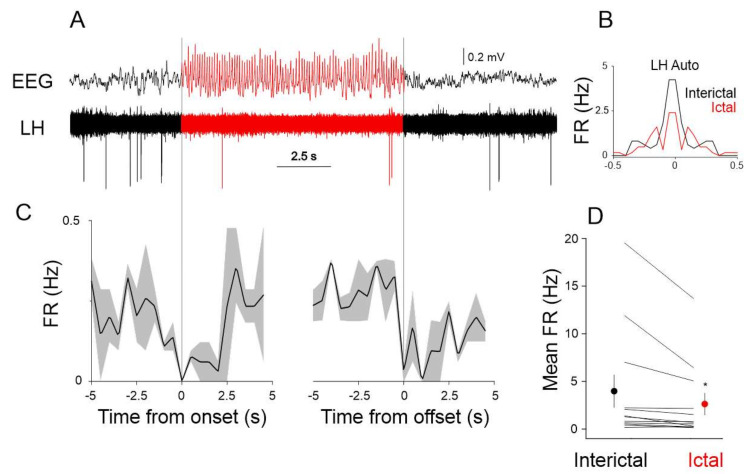
Ictal and interictal neuronal activity in the LH. (**A**) Example single unit recording in the LH during ictal (red) and interictal (black) periods. (**B**) Ictal (red) and interictal (black) autocorrelation of the neuron shown in (**A**), FR: firing rate. (**C**) Peri-event histograms of the firing rate of the neuron shown in (**A**) triggered by the onset (left) and offset of SWDs (see dotted gray lines). Solid lines represent the mean, gray shaded area: s.e.m. (**D**) Mean ictal and interictal firing rates of all the recorded LH neurons (n = 12). Each line represents a neuron, population mean shown as red (ictal) and black (interictal) circles. The asterisk denotes statistical significance.

Given the SWD related local LFP activity in the LH region we next investigated the entrainment of LH neurons to the SWDs. We recorded the single unit activity of LH neurons (n = 12) and correlated it to the SWDs recorded in the S1 using multi-site silicone probes in GAERS rats (Figure 4). When scrutinizing the seizure-related firing dynamics of the recorded LH neurons, we found that the activity of the population of recorded LH neurons significantly changed between ictal (during seizures) and interictal (between seizures) periods (interictal mean firing rate: 3.98 ± 1.73, ictal mean firing rate: 2.63 ± 1.16, *p* < 0.05, Wilcoxon signed-rank test, Figure 4D). 

## 3. Discussion

The present experiments clearly demonstrate that: (i) the LH sends functional monosynaptic excitatory projections to the brainstem DRN; (ii) neuronal activity in LH is modulated by the ongoing seizures both at the population level (LFPs) and single units in the RN; and (iii) the most prominent ictal activity change is a decrease in LH neuronal activity.

We used viral tracing and ChR2-assisted circuit mapping to show that a population of LH neurons establish monosynaptic connections with DRN 5-HT neurons. While previous studies showed that LH GABAergic neurons preferentially contact DRN GABAergic neurons^23^ here we present evidence for a direct excitatory LH to DRN connection mediated through iGluR receptors directed towards 5-HT neurons. The exact neurochemical identity of this subset of DR-projecting LH neurons remains to be established given the numerous subpopulations among hypothalamic neurons [33].

Importantly, in bipolar LFP recordings from the LH area, SWDs of smaller amplitude but similar in shape can be recorded as previously reported^28^. This suggests that the electrical activity of LH neurons is entrained to a certain degree to the ongoing SWDs. Generally, the very low firing rate of most LH neurons recorded does not show evidence of action potential coupling to the individual cycles of the SWDs, but their subthreshold activity might show prominent rhythmic synaptic potentials phase locked to the ongoing SWDs. If this is the case the relatively pronounced SWDs in the LFP could originate from rhythmic SWD coupled inputs. 

Our single unit recordings show a prominent ictal decrease in all the LH neurons recorded. This ictal firing rate reduction will affect the output structures of the LH including the DRN. The consequences of such an ictal decrease in the LH input to the DRN are hard to predict since the neurochemical diversity of both LH and DRN is substantial [33,34], nevertheless, the firing increase observed in putative 5-HT neurons during absence seizures [16] and decreases in identified 5-HT neurons [15] are probably the consequence of LH inputs. Changes in DRN 5-HT neuronal activity can readily alter cortical activity [35], thus the interaction between LH and DRN could be important for the maintenance and termination of seizures. In addition, depression is a common comorbidity associated with epilepsy [36]. It is interesting to note that a recent study found that genetically determined ASs are aggravated, but pharmacologically generated ASs alleviated by antidepressant medication [37] suggesting the relationship between epilepsy and depression is far from simple and awaits further investigations.

## 4. Materials and Methods

All experimental procedures were performed according to the European Communities Council Directives of 1986 (86/609/EEC) and 2003 (2003/65/CE) for animal research and were approved by the Ethics Committee of the University of Szeged. Four male GAERS *rats* were used in this study. The animals were housed in the animal facility of our institute with water and food *ad libitum* in 12 h dark/light cycle with light onset at 7:00 a.m. An effort was made to reduce the suffering and number of animals used. 

For the retrograde labeling of DRN projecting LH neurons 50 nl of PRV-cre virus (a kind gift from Dr. Lynn Enquist) was injected in the DRN of 2 Ai9 *mice* (i.e., tdTomato reporter line [38]). Following 2 months of survival time the animals were anesthetized (200 mg/kg Ketamine, 20 mg/kg Xylazine) and perfused through the heart. Brains were removed and post-fixed in PFA overnight and slices cut at 50 um thickness using a vibrotome (Leica, VT1000, Nussloch, Germany). The slices were mounted on glass slides and covered with coverslips. tdTomato expressing neurons were visualized with a fluorescent microscope (Olympus BX51, Tokyo, Japan, using 4×, 20× and 20× magnification lenses) and counted with respect to their anteroposterior position.

For assessing the effect of local photostimulation of ChR2 expressing LH axons in the DRN we used methods similar to those previously described [23]. Briefly, male WT mice were injected bilaterally with 50 nl of AAV-syn-ChR2-Venus [29] in the LH at the following coordinates (AP: −1.4, ML: 1, DV: −5.2 all mm from Bregma) using a nanoinjector (Nanoliter 2000, WPI, Sarasota, FL, USA) connected to a glass pipette (~20 µm tip diameter) at a rate of 1 nl/s. Following 4 weeks of survival the animals were anesthetized (80 mg/kg Ketamine, 8 mg/kg Xylazine) and perfused through the heart with ice cold cutting solution containing (in mM) 93 *N*-methyl-d-glucamine, 2.5 KCl, 1.2 NaH_2_PO_4_, 30 NaHCO_3_, 20 HEPES, 25 glucose, 5 *N*-acetyl-l-cysteine, 5 Na-ascorbate, 3 Na-pyruvate, 10 MgSO_4_, and 0.5 CaCl_2_. Coronal brainstem slices containing the DRN were cut in the same solution at 4 ºC and for the initial storage of slices (31 ºC for 12 min) following which the slices were stored in a solution containing (in mM) 30 NaCl, 2.5 KCl, 1.2 NaH_2_ PO_4_, 1.3 NaHCO_3_, 20 HEPES, 25 glucose, 5 *N*-acetyl-l-cysteine, 5 Na-ascorbate, 3 Na-pyruvate, 3 CaCl_2_, and 1.5 MgSO at RT. For recording, slices were submerged in a chamber perfused with a warmed (31 °C) continuously oxygenated (95% O_2_, 5% CO_2_) ACSF containing (in mM) 130 NaCl, 3.5 KCl, 1 KH_2_ PO_4_, 24 NaHCO_3_, 1.5 MgSO_4_, 3 CaCl_2_, and 10 glucose. DRN neurons were visualized using standard DIC optics and recorded in whole cell current clamp mode using an EPC9 amplifier (Heka Elektronik, Lambrecht, Germany). Patch pipettes (tip resistance, 4–5 MΩ) were filled with an internal solution containing the following (in mM): 126 K-gluconate, 4 KCl, 4 ATP-Mg, 0.3 GTP-Na_2_, 10 HEPES, 10 kreatin-phosphate, and 8 Biocytin, pH 7.25; osmolarity, 275 mOsm. Access and series resistances were constantly monitored, and data from neurons with a >20% change from the initial value were discarded. Photostimulation was performed through the microscope objective using a blue LED light source (0.5–0.8 mW/mm^2^, Thorlabs, Newton, NJ, USA).

Immunoreactions were performed as previously described [23]. Briefly, after the termination of the in vitro electrophysiological recordings, slices were immersed in 4% PFA in 0.1 M PBS, at 4 °C for 12 h. Coronal sections (50–70 μm) were prepared using a vibratome VT1000S (Leica Microsystems, Nussloch, Germany). The recorded cells were visualized with Cy3-conjugated streptavidin (Jackson ImmunoResearch Laboratories, West Grove, PA, USA) than incubated in primary antibody (anti-GABA-Rb (1:1000, Jackson ImmunoResearch Laboratories, West Grove, PA, USA) and visualized with Dylight649-DARb (1:400, Jackson ImmunoResearch Laboratories, West Grove, PA, USA) secondary antibody, mounted in Vectashield mounting medium (VectorLaboratories, Burlingame, CA, USA) and images taken by a confocal microscope (Olympus FV1000, Tokyo, Japan).

For in vivo recordings, adult GAERS rats were implanted with custom made LFP electrodes (50 um Tungsten, California Fine Wire, Grover Beach, CA, USA) in the S1 (AP: 0.0, ML: 5.5, DV: −1.4 all mm from Bregma) LH (AP: −2.16–2.0, ML: 1.6, DV: −8.0–9.0 all mm from Bregma) for assessing the LFP. For single unit recordings Si probes (32 channel, 4 shank, Neuronexus, Ann Arbor, MI, USA) were implanted in the LH and recorded using an Intan RHD digital amplifier. Single units were isolated using Spike2 software (CED, Cambridge, UK). Electrode tracks were visualized using standard histological procedures.

Data were analyzed offline using Spike2 (CED, Cambridge, UK) and Origin Pro 8.5 (Microcal, Northampton, MA, USA) software. All data are expressed as mean ± s.e.m. unless stated otherwise. Statistical significance was set at 0.05.

## Data Availability

Data is available upon reasonable request from the corresponding author.
